# Maggots Around Colostomy Site: A Case Report

**DOI:** 10.31729/jnma.6656

**Published:** 2021-07-31

**Authors:** Anu Radha Twayana, Neela Sunuwar, Amrit Devkota, Aakrit Dahal, Rabindra Tamang, Kushal Gautam

**Affiliations:** 1 Kathmandu University School of Medical Sciences, Dhulikhel, Nepal; 2 High Care Unit and COVID Care Unit, Nidan Hospital limited, Lalitpur, Nepal; 3 B.P. Koirala Institute of Health Sciences, Dharan, Nepal; 4 Deparment of Oxford University Clinical Research Unit, Patan Academy of Health Sciences, Lalitpur, Nepal; 5 Department of Surgery, Koshi Zonal Hospital, Nepal; 6 Department of Pediatric Research, Patan Academy of Health Sciences, Lalitpur, Nepal

**Keywords:** *colostomy*, *maggots*, *myiasis*, *stoma*

## Abstract

Myiasis is a skin infection caused by developing larvae (maggots) of various Diptera fly species. The two most frequent flies that cause human infestations around the world are Dermatobia hominis (human botfly) and Cordylobia anthropophaga (tumbu fly). Maggots have been found to infest the nose, ear, orbit, tracheostomy wound, face, gums, and serous cavities, among other places. Maggots at the colostomy site are an uncommon occurrence. We report a case of maggots infestation surrounding the colostomy site. We came across a rather rare finding in a patient with advanced inoperable rectum cancer who initially complained of persistent nonspecific pain, discomfort, and foul-smelling discharge from the colostomy site. The issue at hand was identified to be maggots, and their removal alleviated the patient's symptoms. We underline the importance of regularly monitoring stoma sites to avoid maggot infestation, especially in tropical regions.

## INTRODUCTION

Maggot infestation is a parasitic infection caused by dipterous fly larvae, which feed on the living or dead tissues.^1^ It is classified according to the anatomic location or the clinical syndrome.^1^ Fly larvae are called maggots. Its diagnosis is based on visualisation of larvae when superficial and symptoms, when found on internal organs.^2^ Infestation is common in tropical areas including Nepal.^3^ Patients have a variety of risk factors and comorbidities that make them more susceptible to this condition.

## CASE REPORT

A 51 years old female, from Panchthar was admitted to the Surgical Department via the Emergency Department complaining of constant pain over the colostomy site, foul smelling discharge, skin irritation around the colostomy site and sensation of crawling at the site with visible maggots crawling over the site. Prior to the admission she had recently undergone Hartman procedure and loop colostomy for large bowel obstruction secondary to left sided colorectal carcinoma.

**Figure 1:**
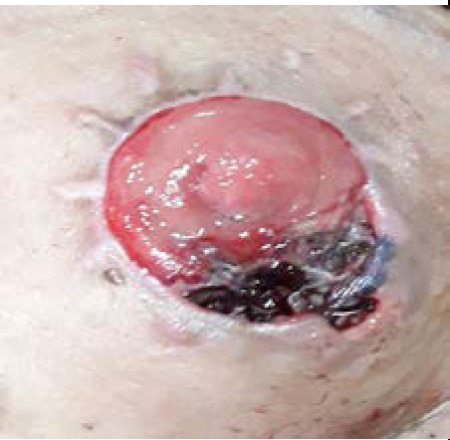
Maggots in the colostomy site at the time of diagnosis

Examination revealed stable vitals with no fever, oedema, pallor and icterus. Her calorie intake was inadequate with a BMI of 18 and her personal hygiene was not maintained. She was anxious and distressed. Her abdominal examination was insignificant except for a sigmoid loop colostomy in the left iliac area. The digital inspection of the lower rectum revealed a hard circumferential growth. Local examination of the colostomy site revealed sloughing over the lower part along with multiple burrows and pus discharge over the skin around the colostomy site. Numerous creeping maggots were found in the burrows while exploration with artery forceps. The skin and subcutaneous tissue are the colostomy site was inflammed (Figure 1).

Laboratory evaluation showed raised white blood cell count and ESR. Other investigations (biochemical and serological) were normal.

**Figure 2:**
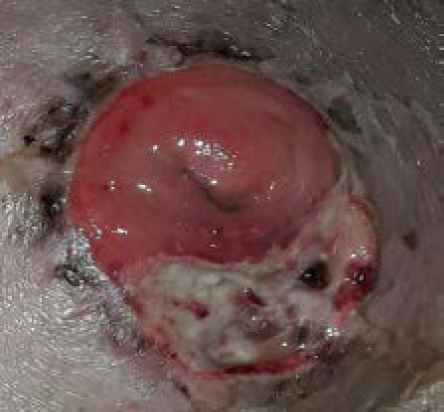
Day 3 of treatment.

The patient was managed conservatively with systemic antibiotics (ceftriaxone and metronidazole) and proper stoma care. The maggot infestation was managed by application of turpentine oil as repellent for maggots followed by gentle removal by instrument. All maggots were removed over 3 days of treatment (Figure 2).

**Figure 3:**
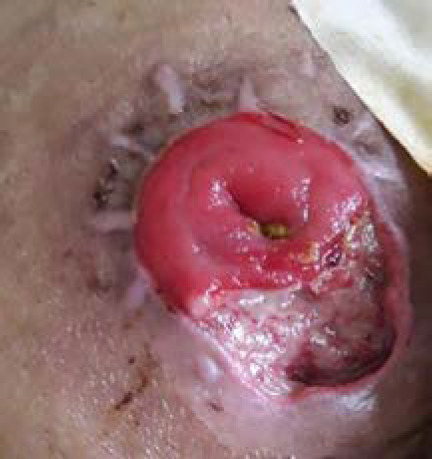
Day 7 of treatment.

Patient was discharged after a week in a healthy condition with a maggot free stoma (Figure 3).

An advice regarding proper colostomy care and palliative treatment for his advanced malignancy of rectum was given on discharge

## DISCUSSION

Myiasis or maggot infestation, a parasitic dipterous fly larvae infestation of tissues is an uncommon wound complication.^1^ The deposition of fly eggs on pus-discharging wounds is the main culprit behind wound myiasis.^2^ It is most common in rural areas of the tropical region especially in patients who have a variety of risk factors and comorbidities that make them more susceptible to this disease.^1^ Colostomy treatment is a critical aspect of post-operative care, but it is often overlooked by patients and their families in developing countries.^3^ Socioeconomic factors such as advanced age, lack of sanitation, prior diabetes, rural housing, homelessness, neurological disorders are linked to disease risk factors.^2^ Debridement, larvae and devitalized tissue removal are often used in conjunction with the administration of Ivermectin and antibiotics.^4^

According to previously published studies, female are drawn to odoriferous suppurating lesions and lay eggs in pre-existing wounds causing majority of cases of human myiasis. The appearance of a ragged, foul-smelling lesion containing maggots is a sign of infestation. Inflammation and toxin are the causes of pathogenicity.^3^

Various authors advocate that occlusion/suffocation methods force the larva to come out, which are then caught with tweezers (or forceps). The occluding materials such as turpentine oil, petroleum jelly, liquid paraffin, beeswax or heavy oil, or lard or bacon strips are usually positioned over the central punctum to coax the larva to emerge head first. We need to be careful not to rupture the maggots, as this could result in secondary infections or a potentially serious allergic reaction.^3^ In this case the usual method of suffocation with turpentine oil was used to force larva to emerge on surface and maggots were removed using artery forceps.

A variety of antibiotic therapies are available and the surgeon's preference is influenced by their experience and drug availability.^2^ The most common drug of choice is ceftriaxone used in conjunction with debridement and removal of necrotic tissue. Subsequently, ivermectin, which belongs to the macrolide family and acts by blocking impulses in the nerve endings by the action of gamma-amino-butyric acid, is also a treatment option for the eradication of the larvae.^2^ However, the patient in this case was treated with ceftriaxone and metronidazole. She was also advised on general supportive measures like improving the nutrition, psychological support and counselling with an emphasis on local hygiene.

Chronic wounds are known to be linked to wound myiasis. There has been a significant association between wound myiasis and diabetes or peripheral vascular disease.^5^ Nonetheless; our patient did not have any underlying diabetes or other significant medical condition. In addition, myiasis is associated with a significant psychological discomfort to the patient and also to the caregiver.^6^ Therefore it is important to address the psychological stress of the patient during treatment. As a result, the patient was referred to psychiatric counselling, which proved to be an excellent decision because it resulted in a large reduction in her distress.

In a tropical country where flies are abundant, it is prudent to include counselling in stoma treatment, and the clinician should recognize it as one of the potential differential diagnoses, particularly in the case of a colostomy presenting with local pain and discomfort. Myiasis is not a condition that must be reported but often cases can go undetected due to cultural and socioeconomic factors. This could be one of the reasons why real myiasis is so uncommon. Therefore, it is important to raise awareness about the condition among general population especially in those with a stoma. Furthermore, health-care professionals must be vigilant to complete mandated reporting, which will aid in determining the true occurrence rate. We suggest the insurgence of the maggot and its effects can simply be avoided with adequate health care, medical vigilance and catering to the psychological needs of the patient.

## References

[1] Waidyaratne G, Zhou S, O'Neil T, Marks A (2021). Management of wound myiasis in the hospice and palliative medicine setting.. J Palliat Med..

[2] Lauand GA, Lima FGGP, Silva RP da, Santiago LR, Dantas JB, Silva CJ (2019). Primary myiasis in surgical wound of mandible symphisis fracture.. Case Rep Pathol.

[3] Jain R, Ranjan V, Dosi R, Nagar S (2013). Insurgence of maggots around colostomy: an unusaul presentation.. Journal of Evolution of Medical and Dental Sciences..

[4] Choudhary V, Choudhary M, Pandey S, Chauhan VD, Hasnani JJ (2016). Maggot debridement therapy as primary tool to treat chronic wound of animals.. Vet World..

[5] Sherman RA (2000). Wound Myiasis in Urban and Suburban United States.. Arch Intern Med..

[6] Asotibe JC, Achebe I, Mbachi C, Igwilo R, Paintsil I (2020). Wound Myiasis in Severe Venous Stasis Ulcer.. Cureus..

